# Complete chloroplast genome sequencing of *Morus alba* var. *pendula* Dippel, 1753 from North China

**DOI:** 10.1080/23802359.2026.2722770

**Published:** 2026-10-21

**Authors:** Binbin Wang, Hui Wang, Danyang Wang, Chunyang Shen, Yanxia Gao

**Affiliations:** a Institute of Sericulture, Chengde Medical University, Chengde, China; b Hebei Sericulture Industry Technology Innovation Center, Chengde, China; c School of Basic Medical Sciences, Chengde Medical University, Chengde, China

**Keywords:** *Morus alba* var. *pendula*, Moraceae, chloroplast genome, phylogenetic analysis

## Abstract

*Morus alba* var. *pendula* Dippel is a widely distributed ornamental mulberry. In this study, we present the complete chloroplast genome of an *M. alba* var. *pendula* accession from North China, which was generated using a combination of Illumina and Oxford Nanopore sequencing data. The circular genome is 158,768 bp long and has a GC content of 36.16%. A total of 129 genes were annotated, which included 85 protein-coding genes, 36 transfer RNA (tRNA) genes, and eight ribosomal RNA (rRNA) genes. Based on 20 chloroplast genomes, phylogenetic analysis demonstrated a close phylogenetic relationship between *M. alba* var. *pendula* and other accessions of the same species, as well as *M. alba* var. “Guoshensang” from Shaanxi Province. The assembled chloroplast genome will substantially benefit future research on phylogeny, population genetics, and conservation in the genus *Morus*.

## Introduction

*Morus alba* var. *pendula* (Moraceae), characterized by its weeping branches, is commonly known as weeping mulberry, “Shidareguwa” in Japan, and “Chuizhisang” in China (Yamanouchi et al. 2009). Widely distributed across tropical to temperate climates, it exhibits strong environmental adaptability, including substantial drought and wind tolerance (Vajna 2000; Jokar et al. 2016; Zeng et al. 2022). Ecologically, it improves stand structure and ecosystem stability in mixed assemblages (Potapenko et al. 2020; Mo et al. 2022). Its dense, curtain-like canopy makes it ideal for landscaping (Vajna 2000), while its rich profile of bioactive compounds (e.g., anthocyanins, phenols, and flavonoids) confers significant medicinal value, including antioxidant, anti-inflammatory, antitumor, and antibacterial properties (Chen et al. 2017, 2018; Wang et al. 2019).

Despite extensive research on its cultivation, *Morus* taxonomy remains challenging due to outcrossing, hybridization and transplantation (Chao et al. 2010; Yang et al. 2014; Sánchez-Salcedo et al. 2015). Recently, two complete chloroplast genomes of *M. alba* var. *pendula* from Shaanxi Province (hereafter SX) and Chongqing City (hereafter CQ), China, were sequenced through Illumina sequencing (GenBank accession PQ594805 and OP153917, respectively) (Zeng et al. 2022; Yang et al. 2025). Nonetheless, comprehensive structural analyses are lacking. Moreover, the chloroplast genome of accessions from North China remains uncharacterized. Here, we combined Illumina and Nanopore sequencing platforms to assemble the complete chloroplast genome of a North China accession. We analyzed its genomic structure and reconstructed a Moraceae phylogeny, providing essential resources for its evolutionary history.

## Materials and methods

### Plant material, DNA extraction, and sequencing

Fresh young leaves of *M. alba* var. *pendula* were collected from Chengde Medical University in Chengde City, Hebei Province, China (41°2′19.40″N, 117°57′48.10″E; hereafter HB) (Figure 1). A voucher specimen was deposited at the Sericultural Research Institute of Chengde Medical University (http://cys.cdmc.edu.cn/, contact: Binbin Wang, wbb232456@cdmc.edu.cn, voucher number: CYYJS02).

**Figure 1.**
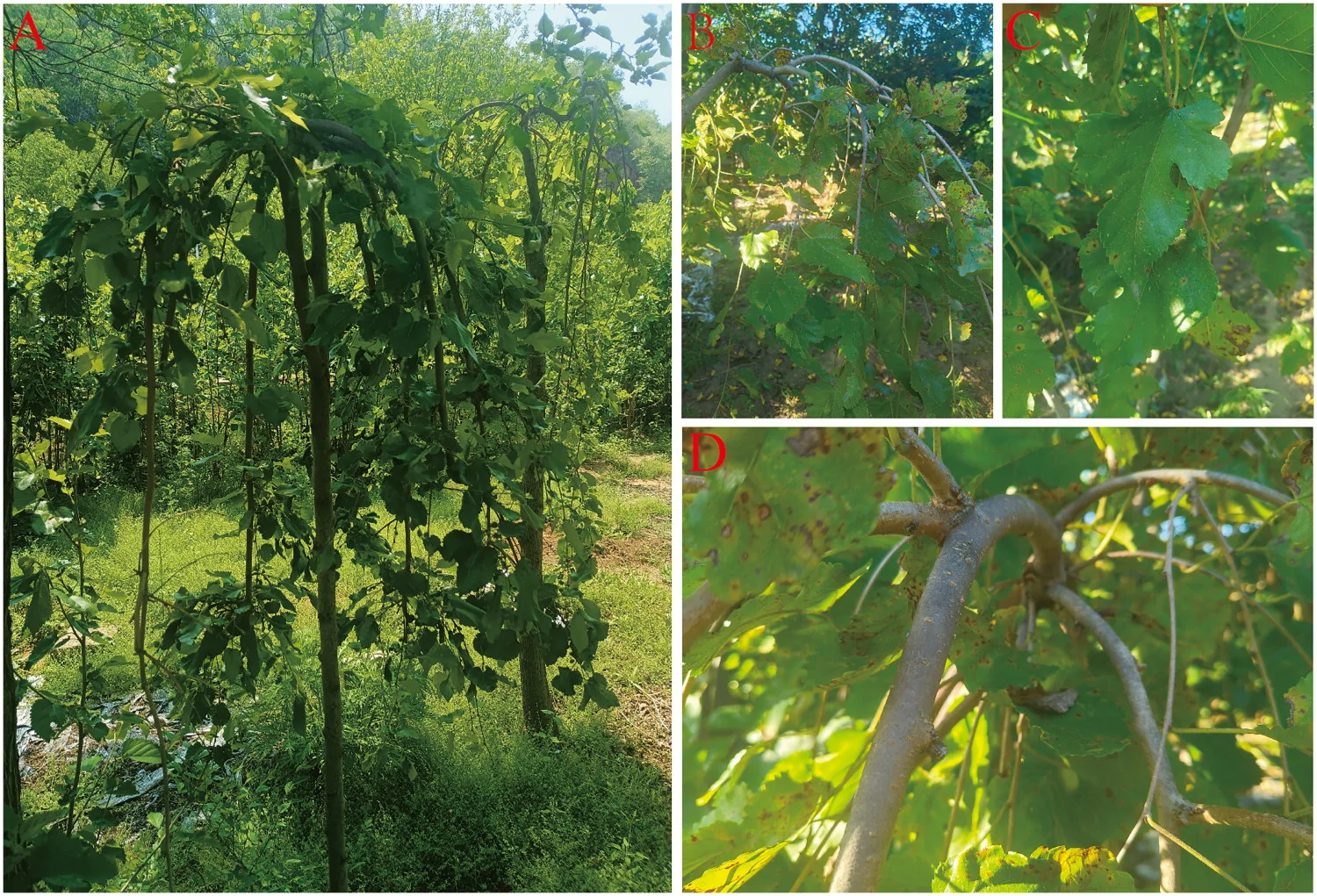
Reference images of HB. Photographs were taken by Hui Wang at Chengde Medical University, Chengde city, Hebei province, China (41°2′19.40″N, 117°57′48.10″E). HB is a perennial shrub or tree with slender, pendulous branches (A, B). The leaves are dark green, lobed, ovate, with obtusely serrate margins, glabrous adaxial surfaces, and pubescent petioles (C). The bark is thick, dark brown, with irregular shallow longitudinal fissures (D).

Total genomic DNA was extracted using a modified cetyltrimethylammonium bromide protocol (Zeng et al. 2022). Before library construction, the purity and integrity of the extracted DNA were assessed. After fragmentation, end repair, A-tailing, and adapter ligation, sequencing libraries for the Illumina short-read platform were generated by Benagen Tech Solutions (Wuhan, China). In parallel, Benagen Tech Solutions (Wuhan, China) simultaneously constructed and sequenced a long-read sequencing library on the Oxford Nanopore Technologies platform. Raw reads were filtered using FastQC v0.11.9 (Cock et al. 2010) and NanoPack2 (Wouter and Rosa 2023).

### Chloroplast genome assembly and annotation

Flye v2.9.2-b1786 was used to de novo assemble long reads using default parameters. Chloroplast contigs were identified using BLASTn searches against *M. indica* and visualized in Bandage v0.8.1 (Wick et al. 2015; Kolmogorov et al. 2019). The preliminary assembly was polished with Illumina reads using Fmlrc2 v0.1.7, Ropebwt2 r187, and Msbwt v0.2.9 (Holt and McMillan 2014; Li 2014; Mak et al. 2023). The genome was annotated with CPGAVAS2 and visualized using OGDRAW (Shi et al. 2019; Stephan et al. 2019). Transfer RNA (tRNA) and ribosomal RNA (rRNA) genes were identified using tRNAscan-SE and BLASTn, respectively (Lowe and Eddy 1997; Chen et al. 2015). Annotations were manually curated using CPGView and Apollo (Lewis et al. 2002; Liu et al. 2023).

### Phylogenetic analysis

To determine the phylogenetic position of HB, 20 plastomes (18 *Moru*s species, HB, and *Camellia neriifolia* as an outgroup) were aligned using MAFFT V7. IQ-TREE V2.4.0 was used to reconstruct a maximum likelihood phylogenetic tree under the K3Pu + F+G4 model (Minh et al. 2020). Branch support was estimated using 1,000 ultrafast bootstrap replicates. The final tree was visualized and edited in iTOL v5.0 (Letunic and Bork 2019).

## Results

Using 5.56 Gb of clean reads, the complete chloroplast genome of HB was assembled and submitted to GenBank with accession number PX243414. The NCBI Sequence Read Archive (accession number SRR34288337) contains the raw sequencing reads. Illumina read mapping achieved a 99.04% rate with no uncovered regions, demonstrating high assembly fidelity (Figure S1). The genome is 158,768  bp long with a 36.16% GC content (Figure 2). It exhibits a typical quadripartite circular structure: a large single-copy (LSC) region (87,861 bp, 33.78% GC), a small single-copy (SSC) region (19,841 bp, 29.20% GC), and two inverted repeat regions (IRA/IRB, 25,533 bp each, 42.96% GC) (Table S1).

**Figure 2.**
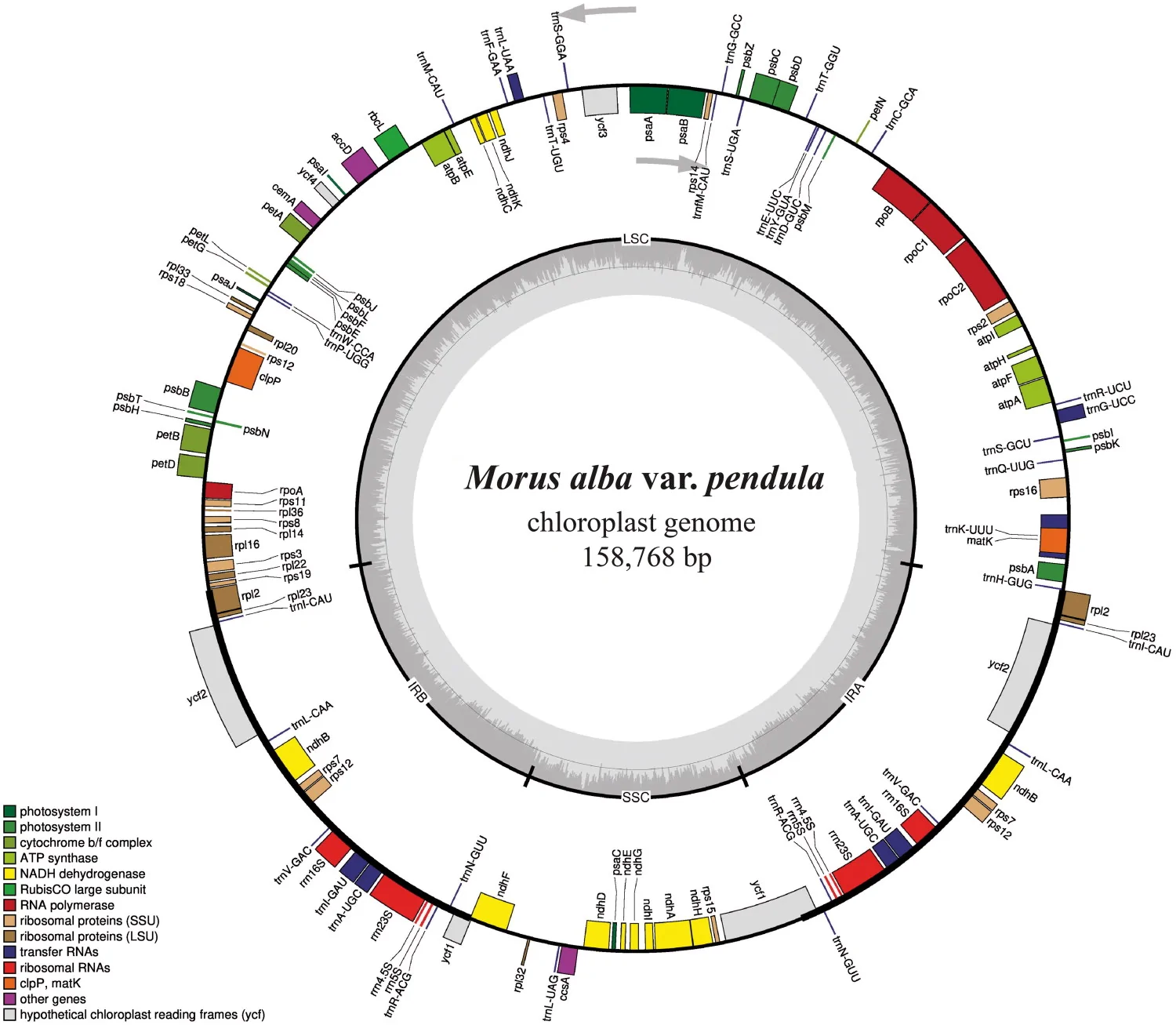
Complete chloroplast genome map of HB comprises three concentric tracks. Genes from different functional groups are plotted on the outer circle. The Middle track shows the genomic structure, comprising the LSC, SSC, and two IR regions. The inner circle shows the overall genome length. Genes are color-coded based on functional classification.

The genome comprises 129 genes, including 85 protein-coding genes, 36 tRNA genes, and eight rRNA genes (Table S2). Most genes are present as single copies, whereas four rRNA genes (*rrn4.5S*, *rrn5S*, *rrn16S*, and *rrn23S*), seven tRNA genes (*trnA-UGC*, *trnI-CAU*, *trnI-GAU*, *trnL-CAA*, *trnN-GUU*, *trnR-ACG*, and *trnV-GAC*), and seven protein-coding genes (*rps12*, *rpl2*, *rpl23*, *rps7*, *ndhB*, *ycf1*, and *ycf2*) are duplicated in the IR regions. Nine *cis*-splicing genes (*rpl2*, *ndhB*, *ndhA*, *rps16*, *atpF*, *rpoC1*, *petB*, *petD*, and *rpl16*) each contain one intron, whereas two genes (*ycf3* and *clpP*) each contain two introns (Figures S2a). The trans-splicing gene *rps12* is also highly conserved (Figures S2b).

Phylogenetic analysis demonstrated that *Morus* forms a monophyletic clade, with HB clustering most closely with SX and *M. alba* “Guoshensang” (Figure 3).

**Figure 3 .**
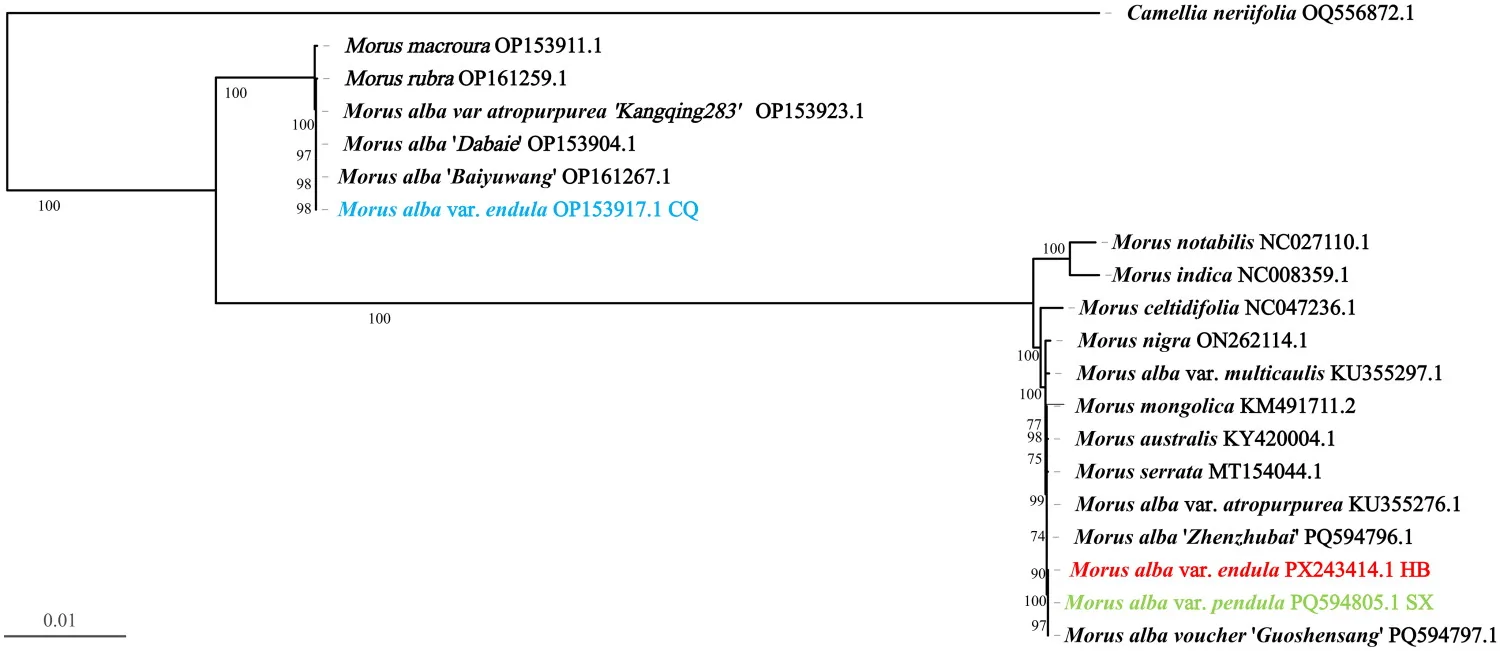
Phylogram of Morus based on complete chloroplast genome sequences. The sequenced HB genome is highlighted in red. The following sequences are used to construct the phylogenetic tree include: *M. alba* var. *pendula* (PX243414.1, HB), *M. mongolica* (KM491711.2) (Ravi et al. 2006), *M. indica* (NC008359.1) (Ravi et al. 2006), *M. alba* var. *multicaulis* (KU355297.1) (Kong and Yang 2017), *M. notabilis* (NC027110.1) (He et al. 2020), *M. alba* var. *atropurpurea* (KU355276.1), *M. alba* var. *pendula* (OP153917.1, CQ) (Zeng et al. 2022), *M. celtidifolia* (NC047236.1) (Zeng et al. 2022), *M. alba* var. *atropurpurea* ‘Kangqing283’ (OP153923.1) (Zeng et al. 2022), *M. alba* var. *pendula* (PQ594805.1, SX) (Zeng et al. 2022), *M. australis* (KY420004.1), *M. serrata* (MT154044.1), *M. rubra* (OP161259.1), *M. nigra* (ON262114.1), *M. alba* ‘Baiyuwang’ (OP161267.1) (Yang et al. 2025), *M. alba* ‘Dabaie’ (OP153904.1) (Yang et al. 2025), *M. alba* ‘Zhenzhubai’ (PQ594796.1) (Yang et al. 2025), *M. alba* ‘Guoshensang’ (PQ594797.1) (Yang et al. 2025), *M. macroura* (OP153911.1) (Zeng et al. 2022), and *Camellia neriifolia* (OQ556872.1) (Ran et al. 2024).

## Discussion and conclusion

We utilized hybrid next-generation (NGS) and third-generation sequencing (TGS) technologies to assemble the chloroplast genome of HB. The HB genome is 516 bp and 525 bp shorter than the previously reported SX and CQ accessions, respectively (Figure S3). NGS generates short reads; therefore, it is challenging to assemble chloroplast genomes with long repetitive sequences or regions with high AT content. In contrast, long reads generated by TGS can overcome these limitations and reduce misassemblies caused by repetitive sequences (Hu et al. 2023). Consequently, chloroplast genomes assembled using NGS data are longer than those assembled using TGS data. Nevertheless, the three genomes share nearly identical gene composition, indicating high conservation of the chloroplast genome.

The length variations among HB, SX, and CQ were primarily located in the IR and LSC regions and could be attributed to insertion and deletion events in intergenic spacer regions or to differences in sequencing technology. Specifically, the IRA and IRB regions of HB were 157 bp shorter than those of SX and CQ. In the LSC region, HB was 200 bp and 210 bp shorter than SX and CQ, respectively (Figure S3). Nevertheless, HB consisted of two additional genes, namely *psbH* and *psbL*, compared with SX and CQ. Of the tRNA genes, only HB comprised *trnS-GGA*, whereas only SX and CQ comprised *trnS-GCU*. According to the sequence alignment of the three chloroplast genomes, the gene order of HB and SX was largely consistent, unlike that of CQ (Figure S4). The SSC region in CQ is inverted compared to other *Morus* genomes, suggesting a potential manual assembly error (Figure S4). This finding may explain why CQ did not cluster in the same clade as HB and SX.

*M. alba var. pendula* is traditionally classified as an *M. alba* variety, supported by our chloroplast phylogeny. However, a simple sequence repeat analysis of 359 mulberry trees showed that *M. alba* var. *pendula* shows a more distant genetic relationship to other mulberry species, including *M. alba* varieties. Thus, it was hypothesized that *M. alba* may represent only one parental lineage of *M. alba* var. *pendula* (Kılınçer et al. 2024). Consequently, its complete genetic background warrants further investigation.

In conclusion, we present a high-quality reference chloroplast genome of *M. alba* var. *pendula* from North China. The genomic and phylogenetic analyses establish a strong basis for future species identification and enrich the genomic resources available for *Morus* taxa.

## Supplementary Material

Table S2 List of genes was annotated in the chloroplast genome of Morus alba var pendula.docx

clean-Supplementary_material.docx

Table S1 Statistical table of chloroplast genome structure of Morus alba var pendula.docx

## Data Availability

The genome sequence data supporting the findings of this study are openly available in the NCBI GenBank repository (https://www.ncbi.nlm.nih.gov/) under the accession number PX243414. The associated BioProject, Sequence Read Archive (SRA), and BioSample numbers are PRJNA1279748, SRR34288337, and SAMN49504156, respectively.
