# Supplementary material for: Complete chloroplast genome sequencing of Morus alba var. pendula Dippel, 1753 from North China
Source: Mitochondrial DNA B Resour. 2026 Oct 21;11(11):1344–8. doi: 10.1080/23802359.2026.2722770 (PMC13689790; doi:10.1080/23802359.2026.2722770)
Supplement: Supplementary file 1 [file TMDN_A_2722770_SM2993.docx]

**Table S1.** Statistical table of chloroplast genome structure of *Morus alba* var. *pendula.*

| Region name | Start | End | Length（bp） | GC content（%） |
| --- | --- | --- | --- | --- |
| LSC | 1 | 87861 | 87861 | 33.78 |
| IR | 87862 | 113394 | 25533 | 42.96 |
| SSC | 113395 | 133235 | 19841 | 29.20 |
| IR | 133236 | 158768 | 25533 | 42.96 |
