# Supplementary material for: Complete chloroplast genome sequencing of Morus alba var. pendula Dippel, 1753 from North China
Source: Mitochondrial DNA B Resour. 2026 Oct 21;11(11):1344–8. doi: 10.1080/23802359.2026.2722770 (PMC13689790; doi:10.1080/23802359.2026.2722770)
Supplement: Supplementary file 2 [file TMDN_A_2722770_SM2994.docx]

**Supplementary material**

**Table S1. of *M*Statistical summary of the chloroplast genome structure *orus alba* var. *pendula***

| Region name | Start | End | Length（bp） | GC content（%） |
| --- | --- | --- | --- | --- |
| LSC | 1 | 87861 | 87861 | 33.78 |
| IR | 87862 | 113394 | 25533 | 42.96 |
| SSC | 113395 | 133235 | 19841 | 29.20 |
| IR | 133236 | 158768 | 25533 | 42.96 |

**Table S2. Annotated genes in the chloroplast genome of *Morus alba* var. *pendula***

| Group of genes | Gene names |
| --- | --- |
| Subunits of NADH-dehydrogenase | *ndh*A，*ndh*B(×2)，*ndh*C，*ndh*D，*ndh*E，*ndh*F，*ndh*G，*ndh*H，*ndh*I，*ndh*J，*ndh*K |
| Subunits of photosystem Ⅰ | *psa*A，*psa*B，*psa*C，*psa*I，*psa*J |
| Subunits of photosystem Ⅱ | *psb*A，*psb*B，*psb*C，*psb*D，*psb*E，*psb*F，*psb*H，*psb*I，*psb*J，*psb*K，*psb*L, *psb*M，*psb*N，*psb*T，*psb*Z，*ycf*3 |
| Subunits of cytochrome b/f complex | *pet*A，*pet*B，*pet*D，*pet*G，*pet*L，*pet*N |
| Subunits of ATP synthase | *atp*A，*atp*B，*atp*E，*atp*F，*atp*H，*atp*I |
| Large subunit of rubisco | *rbc*L |
| Small subunit of ribosome | *rps*2，*rps*3，*rps*4，*rps*7(×2)，*rps*8，*rps*11，*rps*12(×2)，*rps*14，*rps*15，*rps*16，*rps*18，*rps*19 |
| Large subunit of ribosome | *rpl*2(×2)，*rpl*14，*rpl*16，*rpl*20，*rpl*22，*rpl*23(×2)，*rpl*32，*rpl*33，*rpl*36 |
| DNA dependent RNA polymerase | *rpo*A，*rpo*B，*rpo*C1，*rpo*C2 |
| rRNA genes | *rrn*4.5S(×2)，*rrn*5S(×2)，*rrn*16S(×2)，*rrn*23S(×2) |
| tRNA genes | *trn*A-UGC(×2)，*trn*C-GCA，*trn*D-GUC，*trn*E-UUC，*trn*F-GAA，*trn*fM-CAU，*trn*G-GCC，*trn*G-UCC，*trn*H-GUG，*trn*I-CAU(×2)，*trn*I-GAU(×2)，*trn*K-UUU，*trn*L-CAA(×2)，*trn*L-UAA，*trn*L-UAG，*trn*M-CAU，*trn*N-GUU(×2)，*trn*P-UGG，*trn*Q-UUG，*trn*R-ACG(×2)，*trn*R-UCU，*trn*S-GCU，*trn*S-GGA，*trn*S-UGA，*trn*T-GGU，*trn*T-UGU，*trn*V-GAC(×2)，*trn*W-CCA，*trn*Y-GUA |
| Maturase | *mat*K |
| c-type cytochrom synthesis gene | *ccs*A |
| Envelope membrane protein | *cem*A |
| Protease | *clp*P |
| Subunit of Acetyl-CoA-carboxylase | *acc*D |
| Genes of unknown functions Open Reading | *ycf*1(×2)，*ycf*2(×2)，*ycf*4 |

Note: The number in parentheses indicates gene copy number; for example, “×2” indicates two copies of the gene.

**Figure S1 Coverage of the complete chloroplast genome of *M. alba* var. *pendula*.** Clean reads obtained in this study were mapped to the chloroplast genome of *Morus indica* using Bowtie. Coverage depth was calculated using SAMtools and visualized with an R package.

**Figure S2. Schematic diagram of the *cis*-splicing genes (A) and trans-splicing gene *rps12* (B) in the chloroplast genome of *Morus alba* var. *Pendula***. The schematic diagram was generated using CPGView. In panel A, *cis*-splicing genes are shown, with gene names on the left, and gene structures on the right. Exons are shown in black, introns are shown in white. Arrows indicate the transcriptional direction of each gene. In panel B, the trans-spliced gene *rps12* is shown with arrows indicating its orientation. Not that exon lengths are not drawn to scale.

**Figure S3. Comparison of the borders of LSC, IR, and SSC regions in the chloroplast genomes of three *M. alba* var. *Pendula* accessions.** The junctions of LSC/IRb (JLB), IRb/SSC (JSB), SSC/IRa (JSA), and IRa/LSC (JLA) are shown. Genes adjacent to the boundary regions are indicated above or below the main line. This comparison was conducted based on the annotated chloroplast genome structures to assess IR expansion and contraction. The IR boundary comparison was visualized using IRscope.

**Figure S4 Sequence identity plot comparing the chloroplast genomes of the three *M. alba* var. *pendula* accessions.** Whole-genome alignment was performed and visualized using mVISTA program. Gray arrows indicate gene positions and transcriptional direction. Conserved noncoding sequences (CNSs), exons, tRNAs, and rRNAs are shown in pink, dark blue, and light blue, respectively. Sequence similarity is displayed on a 50 – 100% scale.
